# Indications, techniques, and graft survival of mini and corneo-scleral tectonic keratoplasties: A retrospective single-center case series

**DOI:** 10.1371/journal.pone.0289601

**Published:** 2023-08-04

**Authors:** Cornelius Jakob Wiedenmann, Daniel Böhringer, Philip Maier, Thabo Lapp, Katrin Wacker, Sonja Heinzelmann, Thomas Reinhard, Stefan Johann Lang

**Affiliations:** Eye Center, Medical Center, Faculty of Medicine, University of Freiburg, Freiburg im Breisgau, Germany; University of Chieti-Pescara, ITALY

## Abstract

**Purpose:**

Tectonic keratoplasties (TK) are used to treat corneal and scleral perforations and to prevent the loss of the eye. In this study, we retrospectively analyzed indications, surgical procedures, and outcomes of eccentric mini and corneo-scleral tectonic keratoplasties with respect to anatomical survival and clear graft survival rates to identify risk factors for graft failure.

**Methods:**

This retrospective study includes 33 eccentric mini (graft diameter <6 mm) and/or corneo-scleral TK of 32 consecutive patients of a total of 41 TK carried out between 2005 and 2020 in the Eye Center, University of Freiburg, Germany, making up 0.7% of all keratoplasties performed during this period (n = 5557). Patient and graft specific data were extracted from medical files. Anatomical survival—defined as achieving integrity of the globe without further surgical interventions—and clear graft survival—defined as persisting graft clarity—were estimated using the Kaplan-Meier method. We also fitted Cox proportional hazard models to account for factors influencing anatomical and clear graft survival.

**Results:**

Median duration of anatomical success was 72.5 months (95% confidence interval (CI) 18.1—infinite (inf.)) and median duration of clear graft survival was 29.6 months (95% CI 12.5-Inf.). The 1-year survival rate for anatomical survival was 67.6% (95% CI 52.2% - 87.6%) and for clear graft survival 66.4% (95% CI 50.5%– 87.1%). No enucleation was necessary during this time-period. Non-inflammatory primary causes (n = 14) presented a trend towards better anatomical survival rates (median remained above 0.75 during follow-up) compared to inflammatory primary causes (n = 19, median 18.1 months (95% CI 2.8 - inf.)) and longer clear graft survival (median 29.6 months (95% CI 12.5 - inf.) versus 13.1 months (95% CI 3.2 - inf.)). Corneo-scleral grafts (n = 18) compared to corneal grafts (n = 15) showed a trend towards better anatomical survival (more than 50% of eyes did not fail during follow-up period (95% CI 21.9-Inf. months) versus 18.1 months (95% CI 2.4-Inf.)) and clear graft survival (median 29.6 months (95% CI 12.6-Inf.) versus 6.2 months (95% CI 2.8-Inf.)). Old age (n = 11, 75.2 – 90.1 years) compared to young age (n = 11, 6.2 – 60.2 years) was the only hazard ratio (hazard ratio 0.04 (95% CI 0.002–0.8)) that reached the level of significance (p = 0.03).

**Conclusion:**

Eccentric TK is helpful in the successful treatment of a variety of severe eye diseases. Patients at young age, with pre-existing inflammatory conditions or corneal TK are at higher risk for anatomical failure as well as clear graft failure and therefore need to be monitored closely.

## Introduction

The sclera and the cornea are essential for the anatomical and functional integrity of the globe. Defects of these structures lead to perforations, consecutive loss of intraocular tissue, intraocular infection and thus decreased vision, and can finally result in the loss of the affected eye. Therefore, integrity of the sclera and cornea must be restored immediately when defects arise. Bacterial, viral, fungal and acanthamoeba infections as well as autoimmune diseases induce inflammatory defects. Primarily non-inflammatory defects can result from accidental traumatic perforations or after medical procedures i.e. tumor-excisions and erosion of transscleral tubes. There are several techniques for restoring scleral and corneal integrity, including the use of fibrin glue or cyanoacrylate in small defects [1–3], hydrogel-based adhesive patch [4] and amniotic membrane transplantation [5] in slightly larger defects and lamellar keratoplasty [6] and penetrating keratoplasty (PK) [7,8] for large defects. Keratoplasties with the primary goal of restoring the integrity of the globe rather than primarily restoring visual function are referred to as tectonic. Apart from the central emergency keratoplasty with graft diameters of >6.0 mm, for these purposes the eccentric mini-keratoplasty, characterized by a small graft diameter of usually less than 6.0 mm [9], and the corneo-scleral keratoplasty with graft (partially) placed into the sclera and center diverging from optical center of the cornea serve as alternative techniques [10]. While some central grafts become opaque, some (partially) corneo-scleral grafts remain clear. It is of pathophysiologic interest to detect risk-factors contributing to clear graft failure.

Indications, outcomes, and risk factors for graft failure in TK have been described in Caucasian populations [9–11]. However, these analyses were conducted before the introduction of non-corticosteroid immunomodulatory therapy (NCSIT) in high-risk keratoplasties [12–19]. Newer publications in this context have described TK in Asian populations [20,21]. One more recent study in a Caucasian population evaluated the outcome of tectonic eccentric penetrating sclerokeratoplasty à chaud of more than 6 mm diameter as a treatment option for peripheral perforated as well as pre-descemetal corneal ulcerations [11]. Further studies evaluated tectonic keratoplasties using glycerin-cryopreserved grafts [21,22].

Besides all these publications, risk factors that influence the anatomical and clear graft survival, like underlying indications, the use of NCSIT, tectonic graft position and diameter, patient age at surgery and previous keratoplasties have not been investigated. In this study we therefore explore these risk-factors regarding anatomical and clear graft survival of eccentric mini and corneo-scleral TK from an organ culture cornea bank.

## Materials and methods

All consecutive patients who underwent TK at the Eye Center of the University Medical Center Freiburg between 2005 and 2020 were included in this retrospective mono-center cohort study. The study adheres to the Declaration of Helsinki. Approval was given by the local Ethics Committee of the University of Freiburg under the following number (448/20).

### Data collection

Patients receiving eccentric mini (<6 mm) and/or corneo-scleral corneal donor tissue for tectonic indications were included in this analysis. Patients were identified by keyword search in our database. Patients who underwent central penetrating emergency keratoplasty or keratolimbal keratoplasty were excluded, as central penetrating emergency keratoplasties have been characterized before [8] and keratolimbal keratoplasties represent an entity with different physiologic behavior, due to the limbal donor stem cells, that are transferred with the graft [23,24]. If one eye underwent several TK, only the first was included in the analysis. A defined dataset comprising age at surgery, sex, history of previous PK and the primary cause for tectonic graft was extracted from the medical records. To dichotomize the plethora of primary causes for tectonic keratoplasty, they were either allocated towards non-inflammatory or inflammatory diseases. Tumors and traumatic perforations were referred to as non-inflammatory. Defects caused by bacteria, viruses, and auto-immune-inflammation were allocated to the inflammatory group. In addition, graft specific parameters such as endothelial cell density (ECD) of the donor grafts, graft diameter (<4 mm, 4–6 mm, > 6 mm), graft feature (lamellar vs. tectonic) and position of the graft (corneal vs. corneo-scleral) were collected from the surgical reports. Follow-up data included treatment with NCSIT (mycophenolate mofetil (MMF) and/or cyclosporin a (CSA)), graft clarity (clear vs. opaque) and anatomical failure.

### Surgical technique

A decision towards TK instead of amniotic membrane grafts or glue was made, if the perforation was too big to be treated with amniotic membrane grafts. The area to be excised was marked and then cut out using a mechanical trephine. In all cases except one the graft diameter exceeded the recipient bed by 0.25–0.5 mm. In one patient with a lamellar graft an oversize of 1 mm was chosen by the surgeon. As there were large perforations in the recipient’s cornea full thickness grafts were used in all cases. The graft was fixed using 10–0 nylon interrupted sutures and in few cases 7–0 polyglactin interrupted sutures–especially when adapting the graft into parts of the sclera. Corneal grafts were received from the Lions Cornea Bank Baden-Württemberg (Freiburg im Breisgau, Germany); organculture conditions were according to the EEBA standards.[25]. Before October 2012 no minimum ECD was defined. Therefore grafts with ECD below 1000 cells/mm^2^ are included in this analysis. Standard postoperative treatment regime (preservative-free) was as follows: Dexpanthenol eye cream five times daily as long as the epithelial layer is not fully healed. After closure of epithelial defects dexamethasone 1 mg/ml eye drops five times daily (tapered monthly by one drop). Long term treatment with dexamethasone 1 mg/ml eye drops one time daily. Individually, antibiotic, antifungal and antiviral local therapeutics were prescribed additionally.

### Follow-up

Examination data were collected on the day of discharge from the inpatient stay and additionally two weeks after dismissal. Further examinations were scheduled according to the surgeon’s recommendations. NCSIT was administered depending on risk factors for graft rejection and medical contraindications according to the surgeon’s recommendations. Additional appointments were made if complications occurred. Sutures were removed at the discretion of the surgeon or if they became loose or caused inflammation.

### Outcome measure

Anatomical and clear graft failure were independently assessed as primary outcome. Anatomical failure was defined as loss of integrity of the globe requiring additional surgical intervention (amniotic membrane graft or repeated keratoplasty). Clear graft failure was defined as an irreversible loss of graft clarity. Time to graft failure was defined as the first postoperative examination in which the patient presented with a perforation connected to the graft or an opaque graft.

### Study cohort

There were 32 patients who had 41 TK performed on 33 eyes during the study period. One patient with graft versus host disease had five TK on his right eye and three tectonic keratoplasties on the left eye during the study period. Another patient received two consecutive TK on his left eye. One further patient had two TK on his right eye. Patient and clinical characteristics are presented in Table 1 and S1 Table. Frequencies of underlying diseases leading to TK are presented in Table 2. At the time of the first keratoplasty, the median age of the patients was 66.9 years (25% percentile (Q1) 48.9 years, 75% percentile (Q3) 77.4 years). 16 out of 32 patients enrolled were male (50.0%), 16 patients were female (50.0%). In 20 of 33 cases (60.6%) the right eye and in 13 cases (39.4%) the left eye was affected. The main cause of corneal impairment requiring TK was inflammatory in 19 eyes (57.6%) vs. non-inflammatory causes in 14 eyes (42.4%) (for further details see Table 2). The position of graft placement was corneal in 15 eyes (45.5%) vs. corneo-scleral in 18 eyes (54.6%). The median graft diameter was 5.5 mm (Q1 4.0 mm, Q3 6.2 mm). 26 of 33 cases (78.8%) did not receive NCSIT while seven patients (21.2%) received NCSIT with MMF and/or CSA. Median follow up was 10 months (Q1 3.9 months, Q3 29.6 months). No primary graft failures were observed. No eye had to be enucleated during the follow-up period. TK as defined above (n = 41) made up 0.7% of all keratoplasties including lamellar keratoplasties (n = 5557) and 1.5% of all penetrating keratoplasties (n = 2652) performed at our eye center between 2005 and 2020. In comparison, 165 central emergency keratoplasties were performed during this time period.

**Table 1:** Overall patient and clinical characteristics of all included patients at first TK at our eye center.

	Overall (N = 33)
**Sex**	
female	16 (48.5%)
male	17 (51.5%)
**Eye**	
right	20 (60.6%)
left	13 (39.4%)
**Age at surgery (y)**	
Median [Q1, Q3]	66.9 [48.9, 77.4]
**Follow up (m)**	
Median [Q1, Q3]	10.0 [3.9, 29.6]
**Diameter of graft (mm)**	
Median [Q1, Q3]	5.5 [4.0, 6.2]
**Primary cause**	
non-inflammatory	14 (42.4%)
inflammatory	19 (57.6%)
**Site of graft placement**	
corneal	15 (45.5%)
corneo-scleral	18 (54.5%)
**Graft**	
lamellar	7 (21.2%)
full thickness	26 (78.8%)
**Systemic immunosuppression**	
no	26 (78.8%)
yes	7 (21.2%)
**Endothelial cell density of graft (cells/mm^2^)**	
Median [Q1, Q3]	1898 [1460, 2117]

**Table 2:** Frequencies of underlying diagnoses for tectonic keratoplasties.

**Non-inflammatory indications**	
**Frequency**	**Diagnosis**
**2x**	Limbal cyst
**1x**	Fuchs adenoma of ciliary body
**1x**	Epithelial downgrowth with cystic formation
**1x**	Iris melanoma
**1x**	Limbal hyperpigmentation
**1x**	Conjunctival melanosis
**1x**	Traumatic corneal perforation
**1x**	Epithelial downgrowth after IOL-explantation
**2x**	Perforating foreign body
**1x**	Spontaneous corneal perforation in toxic epidermal necrolysis
**2x**	Tube erosion after Ahmed-valve implant
**Inflammatory indications**	
**Frequency**	**Diagnosis**
**2x**	Rheumatoid arthritis
**2x**	Chronic graft-versus-host-disease
**2x**	Atopic dermatitis
**4x**	Herpetic keratitis
**1x**	Perforated corneal descemetocele after PK
**1x**	Ulcer due to spike thread after scleral fixation of IOL
**1x**	Ocular cicatricial pemphigoid
**6x**	Unknown origin of which four had previous PK

### Statistical analysis

Statistical analysis included descriptive reporting, where the median and 25%/75% percentiles (Q1 and Q3) were calculated for the continuous variables; frequency distribution and percentages were used for categorical variables. For the statistical inference, only the first keratoplasty performed in our hospital was included. Kaplan–Meier survival curves were used to estimate graft survival and to study the aforementioned putative risk factors for graft failure. Categorical variables were analyzed graphically by means of plotting survival curves for the factor levels and reporting median (95% confidence interval (CI)) survival as well as 1-year survival rates. Risk factors for graft failure were analyzed using Cox-regression, represented as hazard ratios with the 95% confidence intervals. We built the final model (Fig 4) by means of stepwise selection. P < 0.05 was considered statistically significant. We did not correct for multiple testing. The R Software version 4.0.1 and RStudio Software version 1.2.5042 were used to analyze the data [26].

## Results

**Fig 1:**
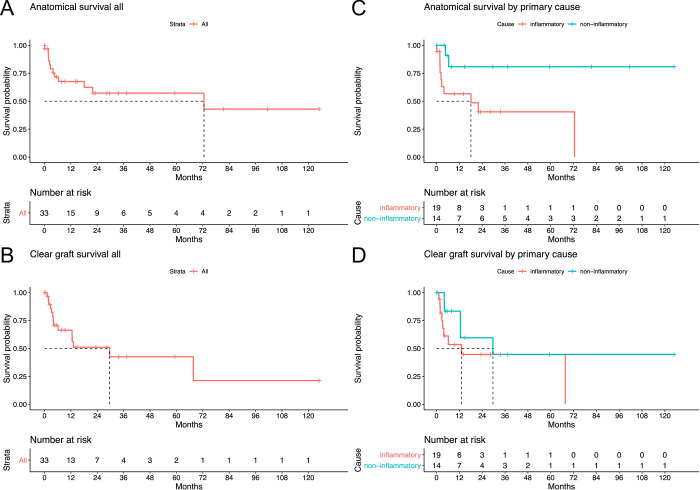
A&B: Kaplan-Meier survival curve of all TK performed during the study period. The 1-year survival rate for anatomical survival was 67.6% (95% CI 52.2% - 87.6%) and for clear graft survival 66.4% (95% CI 50.5%– 87.1%). C&D: Kaplan-Meier estimation for inflammatory and non-inflammatory causes. The median for anatomical survival for inflammatory causes was 18.1 months (95% CI 2.8 - inf.). The median anatomical survival for non-inflammatory causes remained above 0.75 during follow-up. The 1-year survival rate of anatomical survival was 56.7% (95% CI 36.9% - 86.9%) for inflammatory and 80.8% (95% CI 60.0% - 100%) for non-inflammatory causes. Median clear graft survival for inflammatory causes was 13.1 months (95% CI 3.2 - inf.). Median clear graft survival for non-inflammatory causes was 29.6 months (95% CI 12.5 - inf.). The 1-year survival rate of clear graft survival was 53.5% (95% CI 33.2% - 86.4%) for inflammatory and 83.3% (95% CI 64.7% - 100%) for non-inflammatory causes.

In four of 33 eyes, more than one tectonic keratoplasty was necessary to achieve final anatomical success. Median anatomical survival was 72.5 months (95% CI 18.1 - inf.) and clear graft survival 29.6 months (95% CI 12.5 - inf.) after surgery (Fig 1A and 1B). Of note, none of the patients with tumor presented a relapse during follow-up. Comparing Kaplan-Meier estimation of ocular diseases with underlying inflammation showed a trend towards shorter anatomical and clear graft survival compared to eyes with non-inflammatory conditions. The difference was more pronounced in anatomical survival than in clear graft survival (Fig 1C and 1D). Photographs of exemplary cases are presented in S2–S5 Figs.

**Fig 2:**
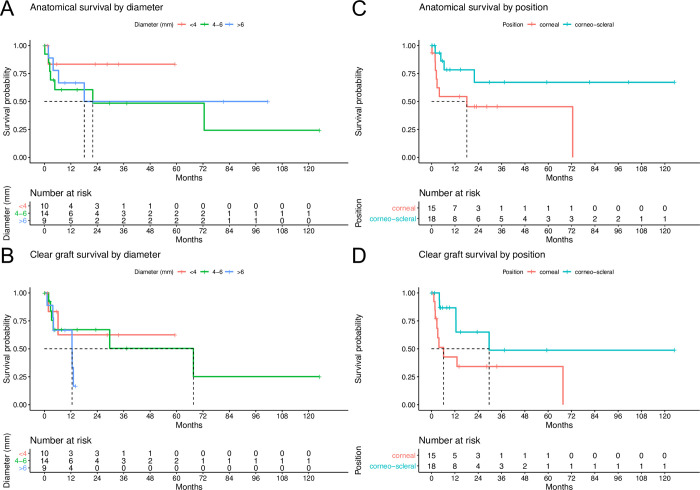
A&B: Kaplan-Meier survival curves for grafts of different diameters. Median anatomical survival of grafts of 4–6 mm diameter was 21.9 months (95% CI 2.8 - inf.) and for nine grafts >6 mm diameter 18.1 months (95% CI 6.4 - inf.). The 1-year survival rate for anatomical survival was 83.3% (95% CI 58.3% - 100%) for grafts <4 mm, 60.6% (95% CI 38.7% - 94.7%) for grafts 4–6 mm and 66.7% (95% CI 42.0% - 100%) for grafts >6 mm. Median clear graft survival for 14 grafts of 4–6 mm diameter was 2.2 months (95% CI 0.1 – inf.) and for nine grafts >6 mm diameter 12.5 months (95% CI 4.1 - inf.). The 1-year survival rate for clear graft survival was 62.5% (95% CI 32.0% - 100%) for grafts <4 mm, 67.1% (95% CI 45.3% - 99.6%) for grafts 4–6 mm and 66.7% (95% CI 42.0% - 100%) for grafts >6 mm. C&D: Median for anatomical survival of solely corneal grafts was 18.1 months (95% CI 2.4 - inf.). In eyes with corneo-scleral grafts more than 50% of eyes showed anatomical survival during the follow-up period (95% CI 21.9 - inf. months). The 1-year survival rate for anatomical survival was 54.4% (95% CI 33.1% - 89.5%) for corneal grafts and 78.3% (95% CI 59.3% - 100%) for corneo-scleral grafts. Median for clear graft survival for solely corneal grafts was 6.2 months (95% CI 2.8 - inf.) and 29.6 months (95% CI 12.6 - inf.) for corneo-scleral grafts. The 1-year survival rate for clear graft survival was 42.7% (95% CI 22.2% - 82.3%) for corneal grafts and 86.7% (95% CI 71.1% - 100%) for corneo-scleral grafts.

Small grafts showed a trend towards superior anatomical survival than medium and large grafts (Fig 2A). Regarding clear graft survival, small and medium grafts showed a trend towards superior survival compared to large grafts (Fig 2B). To evaluate the effect of the position of the graft in the recipient’s eye on graft survival, a survival analysis comparing corneal vs. corneo-scleral grafts was performed. Corneal grafts showed a trend towards inferior anatomical and clear graft survival compared to corneo-scleral grafts (Fig 2C and 2D). Of note: 13 of 18 corneo-scleral grafts (72.2%) had non-inflammatory primary causes compared to one out of 15 of corneal grafts (6.7%).

We investigated the role of age regarding graft survival by splitting our study cohort into three equally sized groups containing 11 patients, respectively. The groups consisted of patients below the age of 60.2, of patients between 60.2 and 75.2, and patients of more than 75.2 years of age. Seven (63.6%) patients with inflammatory conditions were in the group below 60.2 years, three (27.3%) in the group between 60.2 and 75.2 years and nine (81.8%) in the group above 75.2 years. Patients below the age of 60.2 years presented a trend towards inferior survival compared to older patients. To analyze the relevance of previous keratoplasties for graft survival we compared patients who had no previous PK to those who had at least one PK in the past. Anatomical survival in eyes with previous PK showed a trend towards inferiority compared to eyes without previous PK. Eyes with previous PK showed a trend towards shorter clear graft survival compared to patients without previous PK (S1C and S1D Fig). Comparing the survival rates of lamellar and full thickness grafts, no significant differences of anatomical and clear graft survival was found (S1E and S1F Fig).

**Fig 3:**
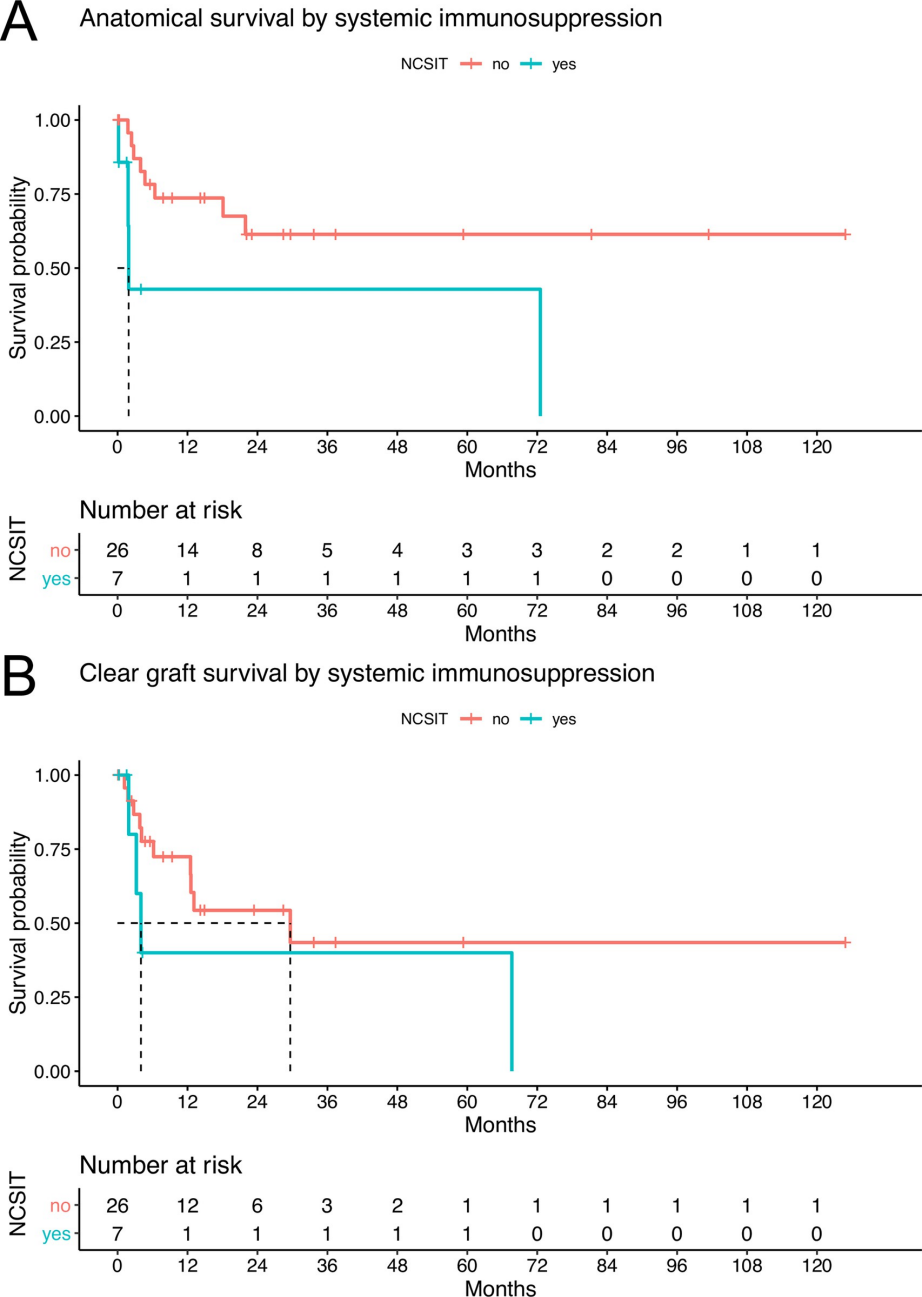
Kaplan-Meier estimates of grafts in patients with or without NCSIT. Median anatomical survival of grafts in patients with prescribed NCSIT was 1.9 months (95% CI 1.8 - inf.). Anatomical survival of grafts in patients without NCSIT remained above 50% during the follow-up period (95% CI 21.9 - inf. months). The 1-year survival rate for anatomical graft survival was 42.9% (95% CI 15.4% - 100%) for patients with NCSIT and 73.6% (95% CI 57.6% - 94.2%) for patients without NCSIT. Median clear graft survival of patients without NCSIT was 29.6 months (95% CI 12.5 - inf.). Median clear graft survival in patients with NCSIT was 4.0 months (95% CI 3.2 - inf.). The 1-year survival rate for clear graft survival was 40.0% (95% CI 13.7% - 100%) for patients with NCSIT and 72.4% (95% CI 55.8% - 94.0%) for patients without NCSIT.

Grafts from patients prescribed NCSIT showed a trend towards inferior anatomical and improved clear graft survival compared to grafts from patients without NCSIT (Fig 3).

**Fig 4:**
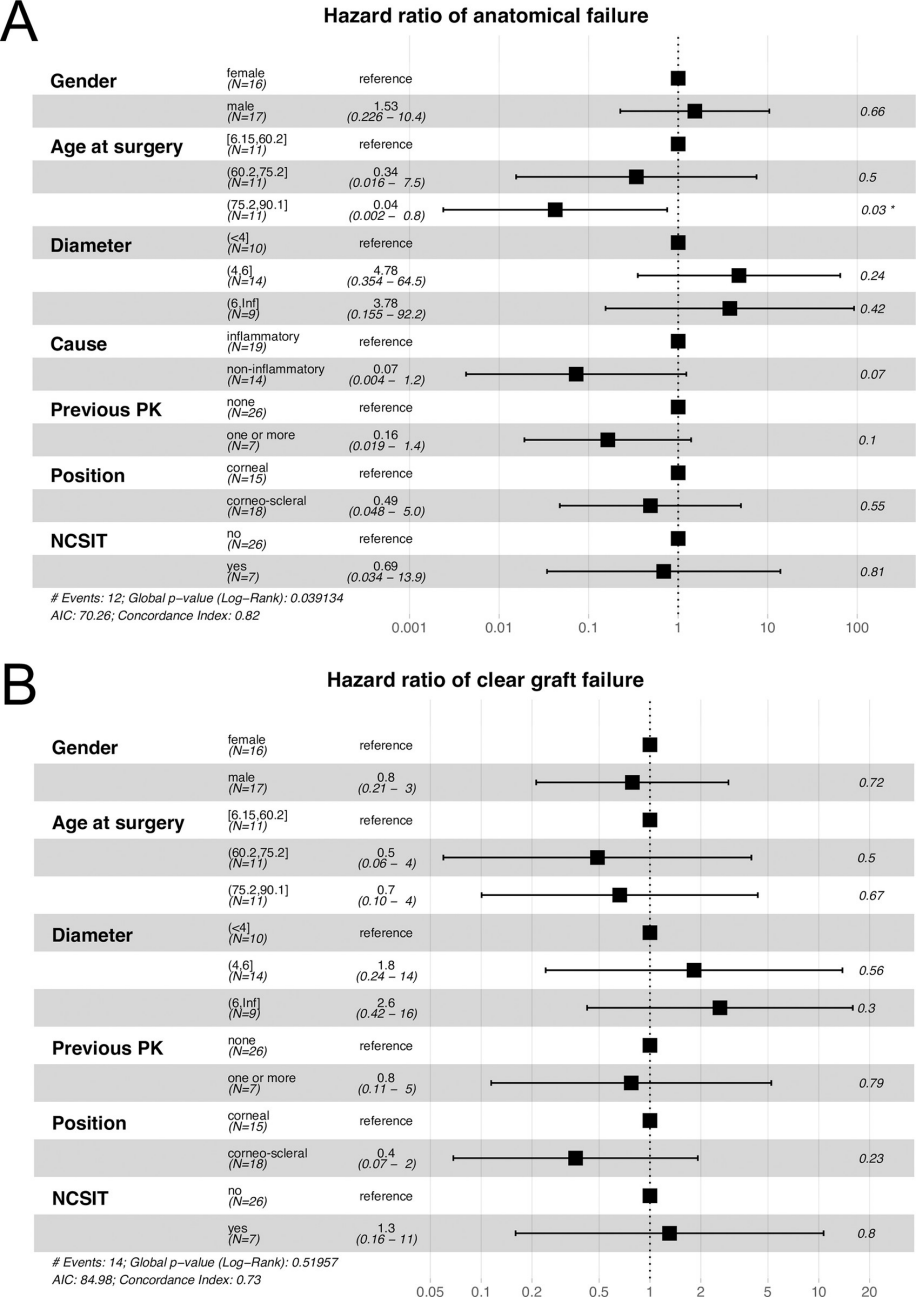
Hazard ratios for patient and graft specific parameters. Hazard ratio and confidence interval are displayed numerically and graphically. P-values are as indicated in the right column. Hazard ratios <1 indicate longer graft survival than the reference group. Hazard ratios >1 indicate shorter graft survival than the reference group.

Cox proportional hazards were calculated for anatomical failure and clear graft failure. The hazard ratio of anatomical failure was statistically significantly lower (HR = 0.04, P = 0.03, 95% CI 0.002–0.8) in patients older than 75 years compared to patients younger than 60 years (Fig 4 and S1A Fig). The hazard ratios of all other categories showed only a trend towards longer or shorter graft survival, but remained below the level of significance.

### Discussion

Mini or corneo-scleral tectonic keratoplasty are surgical options for patients with (impending) anatomical failure and risk of a loss of the eye in cases that would require very large central (keratolimbal) grafts and eyes where cyanoacrylate, fibrin glue, or amniotic membrane are not expected to offer sufficient support to restore globe integrity. In these situations, the primary goal of TK is to preserve the globe, not to restore vision.

In this study, all eyes were preserved by TK during follow-up. No eyes were lost because of enucleation or evisceration. Thus, TK is a sufficient method to achieve preservation of the globe.

Regarding anatomical survival and clear graft survival, subgroups with a trend towards increased risk were identified. Inflammatory primary causes showed a trend towards increased risk of anatomical and clear graft failure compared to non-inflammatory primary causes. Large grafts showed inferior clear graft survival and medium and large grafts showed a trend towards shorter anatomical success compared to smaller grafts. Corneo-scleral grafts presented a trend towards better anatomical survival and clear graft survival compared to solely corneal grafts. Younger patients presented significantly shorter anatomical survival and a trend towards inferior clear graft survival. Patients with previous PK showed a trend towards shorter anatomical and clear graft survival than patients without previous PK. The results are discussed below.

Due to the retrospective nature of our study, the follow-up period was highly variable. However, with a median follow-up of 10.0 months our study is absolutely comparable to other studies [10,20,21]. This is the first study investigating risk factors for clear graft failure and anatomical failure in the age of NCSIT for high-risk keratoplasties for corneal and corneo-scleral grafts. The investigations by Riedel et al. and Vanathi et al. did not include patients receiving NCSIT [10,21]. The study by Ang et al. did not include corneo-scleral grafts and primarily evaluated penetrating versus anterior lamellar keratoplasties.

Age at surgery among patients varied between 6 and 90 years of age. With only 11 of 33 patients being under 60 years of age, it is nevertheless a surgery more often performed in elderly patients. In a study from India, patients were considerably younger with a mean age of 38 years at surgery [21]. Ang et al. from Singapore report a mean age of 51 years, Völcker and Naumann from Germany of 54 years and Kohlhaas et al. from Germany of 63 years [9,11,20]. Interestingly, anatomical success was significantly better in the oldest third of patients than in the youngest third of patients although the prevalence of non-inflammatory and inflammatory conditions was similar in these groups (Fig 4 and S1 Fig). Furthermore, age at surgery was the only risk factor, that reached the level of significance at the cox proportional hazard. Therefore, younger age appears to be a risk factor for graft failure.

Only nine of 33 grafts were more than 6 mm in diameter. Thus, the classification as mini-keratoplasty (2 to 6 mm in diameter) applies to the majority of the tectonic grafts included in this study [9]. Small diameters are preferred in peripheral lesions as the optical axis might be spared and thus visual outcomes can be superior compared to larger grafts affecting the central cornea. However, as demonstrated in patient number nine (S4 Fig), even mainly scleral grafts sometimes require large diameters. Regarding clear graft survival and anatomical survival, smaller grafts showed a trend towards superior survival compared to larger grafts. Smaller grafts are potentially less likely to trigger an immune-response compared to larger grafts. Smaller grafts might also be the choice in smaller corneal lesions, so they are potentially associated with less severe corneal diseases. Therefore, if feasible, smaller grafts should be preferred over larger grafts.

Several other studies primarily focused on inflammatory ocular surface disease as indications of tectonic eccentric keratoplasties [11,27]. In our study 42.4% of cases were non-inflammatory eye diseases. This shows that tectonic eccentric keratoplasty has an important role beyond treatment of inflammatory eye diseases. Especially for en bloc excision of tumors located in or near the anterior chamber angle, this technique is of crucial importance [28]. In certain cases, a clear corneo-scleral graft enables onsight examination for tumor recurrence. Patients with non-inflammatory ocular surface diseases also presented a reduced hazard ratio of tectonic failure. Other studies have shown an inferior outcome of penetrating keratoplasty in acute inflammatory diseases as well [8,29]. This is likely caused by alteration of the immune privilege subsequent to inflammation of the anterior ocular segment.

Besides acute inflammation several other risk factors for graft failure are known. These include previous infectious disease like herpes-keratitis [30], having deep vascularization in host cornea in three or four quadrants [17], a history of previous keratoplasty, positioning of the graft close to the limbus, severe atopic dermatitis [31], steroid-response glaucoma, limbo-keratoplasty, limbal-stem-cell insufficiency and keratoplasty in newborns. In our study one out of five patients received NCSIT with CSA or MMF. NCSIT is known to improve results after high-risk keratoplasties by treating the underlying disease [12–14]. In our retrospective analysis only one of seven patients receiving NCSIT was followed for more than 12 months. During the short follow-up, patients with NCSIT presented a trend towards reduced clear graft and anatomical survival compared to patients without NCSIT. This is rather attributable to the increased risk already existing at the time of transplantation and should not lead to the conclusion that NCSIT has no benefit.

In our study, tectonic keratoplasties showed median clear graft survival of 29.6 months and anatomical success of 72.5 months. Increasing eccentricity of the graft does not necessarily seem to have an unfavorable effect on graft opacity. This has been reported by Riedel et al. and was also apparent in our study [10]. A positive trend for corneo-scleral grafts was also found regarding anatomical success in our patient cohort. This is likely related to the higher percentage of non-inflammatory primary causes in corneo-scleral grafts.

The limitations of our study are the small number of cases and the retrospective approach. However, it would be difficult to perform a randomized controlled trial for such difficult clinical situations because of the rare performance of this surgery and the plethora of underlying diseases. We therefore intentionally included patients with all diagnoses, which reduced the specificity of our results compared with a study of patients with a single entity. Due to the broad range of possible ocular surface disease, choosing a single entity would have neglected the nature of tectonic keratoplasties.

In summary, mini and eccentric TK reflect a small group among all keratoplasty techniques, but in specific situations this surgical technique is the only or best possible option to achieve anatomical integrity of the globe and to avoid enucleation or evisceration. The ultimate goal to avoid enucleation or evisceration was achieved in all cases, but some patients required further surgical interventions. Our analysis shows satisfying results regarding clear graft survival and anatomical success. Thus, mini and corneo-scleral tectonic keratoplasties are of great value in a plethora of ophthalmic diseases.

## Supporting information

S1 Fig Kaplan-Meier survival curves for different recipient age categories, patients with and without previous PK and lamellar vs. full-thickness TK. A&B: Kaplan-Meier survival curves for different recipient age categories. Median anatomical survival of grafts in patients below 60.2 years of age was 18.1 months (95% CI 1.8 - inf.). The 1-year survival rate for anatomical survival was 50.5% (95% CI 27.3% - 93.3%) for patients <60.2 years of age, 69.3% (95% CI 45.3% - 100%) for patients 60.2–75.2 years of age and 85.7% (95% CI 63.3% - 100%) for patients >75.2 years of age. Median clear graft survival for 11 grafts in patients below 60.2 years of age was 12.5 months (95% CI 3.2 - inf.). Clear graft survival of grafts in older patients remained above 50% during follow-up (95% CI 2.8 - inf. months). The 1-year survival rate for clear graft survival was 60.0% (95% CI 36.2% - 99.5%) for patients <60.2 years of age, 80.0% (95% CI 58.7% - 100%) for patients 60.2–75.2 years of age and 58.3% (95% CI 31.3% - 100%) for patients >75.2 years of age. C&D: Kaplan-Meier estimates for eyes with and without previous PK. Median anatomical survival in patients with one or more previous PK was 18.1 months (95% CI 3.9 - inf.). In eyes without previous PK, more than 50% of eyes showed anatomical survival during the follow-up period (95% CI 6.4 - inf. months). The 1-year survival rate for anatomical graft survival was 71.4% (95% CI 44.7% - 100%) for patients with previous PK and 66.7% (95% CI 49.3% - 90.2%) for patients without previous PK. Median clear graft survival of eyes without previous PK was 6.2 months (95% CI 3.8 - inf.) and 29.6 months (95% CI 12.6 - inf.) for eyes without one or more previous PK. The 1-year survival rate for clear graft survival was 38.1% (95% CI 13.7% - 100%) for patients with previous PK and 75.8% (95% CI 59.4% - 96.8%) for patients without previous PK. E&F: Kaplan-Meier estimates for eyes with lamellar and full thickness TK. In eyes with lamellar TK, more than 50% of eyes showed anatomical survival during the follow-up period (95% CI 22.0 - inf. months). Median anatomical survival in patients with full thickness TK was 72.6 months (95% CI 6.4 - inf.). The 1-year survival rate for anatomical survival was 83.3% (95% CI 58.3% - 100%) for patients with lamellar TK and 64.9% (95% CI 48.0% - 87.8%) for patients with full thickness TK. Median clear graft survival in patients with lamellar TK was 6.2 months (95% CI 4.0 - inf.). Median clear graft survival in patients with full thickness TK was 29.7 months (95% CI 12.5 - inf.). The 1-year survival rate for clear graft survival was 40.0% (95% CI 13.7% - 100%) for patients with lamellar TK and 73.4% (95% CI 57.2% - 94.2%) for patients with full thickness TK.
(PDF)

S2 Fig Patient ID 2 16 weeks post-surgery after treatment of a perforated corneal ulcer. (JPG)

S3 Fig Patient ID 28 A: Patient with ciliary-body/iris-melanoma one month and 22 days pre-surgery, B: 27 days post-surgery, C: Three years and four months post-surgery.
(PDF)

S4 Fig Patient ID 9 A: 20 days pre-surgery, presenting conjunctival melanosis, corneal thinning and deep corneal vascularization after radiation of a pigmented tumor in this area 40 years before at a different hospital. B: 4 months post-surgery, C: Three years and five months after initial tectonic keratoplasty and after repeated central keratoplasty because of recurrent corneal ulcer.
(PDF)

S5 Fig Patient ID 15 A: Pre-surgery: Epithelial downgrowth with cystic formation after explosive trauma as a child, B: One day post-surgery, C: Six years post-surgery.
(PDF)

S1 File Raw data. (XLSX)

S1 Table Individual patient and clinical characteristics of all included patients at first TK at our eye center. ID 19 and 24 correspond to the right and left eye from the same patient. The width of the blue bar at the columns age at surgery, diameter and follow up (FU) are proportional to the numbers displayed. In the endothelial cell density (ECD) column, yellow represents low and blue high ECD.
(DOCX)
