# Supplementary material for: Indications, techniques, and graft survival of mini and corneo-scleral tectonic keratoplasties: A retrospective single-center case series
Source: PLoS One. 2023 Aug 4;18(8):e0289601. doi: 10.1371/journal.pone.0289601 (PMC10403125; doi:10.1371/journal.pone.0289601)
Supplement: S1 Fig — A&B: Kaplan-Meier survival curves for different recipient age categories. Median anatomical survival of grafts in patients below 60.2 years of age was 18.1 months (95% CI 1.8 - inf.). The 1-year survival rate for anatomical survival was 50.5% (95% CI 27.3% - 93.3%) for patients <60.2 years of age, 69.3% (95% CI 45.3% - 100%) for patients 60.2–75.2 years of age and 85.7% (95% CI 63.3% - 100%) for patients >75.2 years of age. Median clear graft survival for 11 grafts in patients below 60.2 years of age was 12.5 months (95% CI 3.2 - inf.). Clear graft survival of grafts in older patients remained above 50% during follow-up (95% CI 2.8 - inf. months). The 1-year survival rate for clear graft survival was 60.0% (95% CI 36.2% - 99.5%) for patients <60.2 years of age, 80.0% (95% CI 58.7% - 100%) for patients 60.2–75.2 years of age and 58.3% (95% CI 31.3% - 100%) for patients >75.2 years of age. C&D: Kaplan-Meier estimates for eyes with and without previous PK. Median anatomical survival in patients with one or more previous PK was 18.1 months (95% CI 3.9 - inf.). In eyes without previous PK, more than 50% of eyes showed anatomical survival during the follow-up period (95% CI 6.4 - inf. months). The 1-year survival rate for anatomical graft survival was 71.4% (95% CI 44.7% - 100%) for patients with previous PK and 66.7% (95% CI 49.3% - 90.2%) for patients without previous PK. Median clear graft survival of eyes without previous PK was 6.2 months (95% CI 3.8 - inf.) and 29.6 months (95% CI 12.6 - inf.) for eyes without one or more previous PK. The 1-year survival rate for clear graft survival was 38.1% (95% CI 13.7% - 100%) for patients with previous PK and 75.8% (95% CI 59.4% - 96.8%) for patients without previous PK. E&F: Kaplan-Meier estimates for eyes with lamellar and full thickness TK. In eyes with lamellar TK, more than 50% of eyes showed anatomical survival during the follow-up period (95% CI 22.0 - inf. months). Median anatomical survival in patients with full thick [file pone.0289601.s001.pdf]

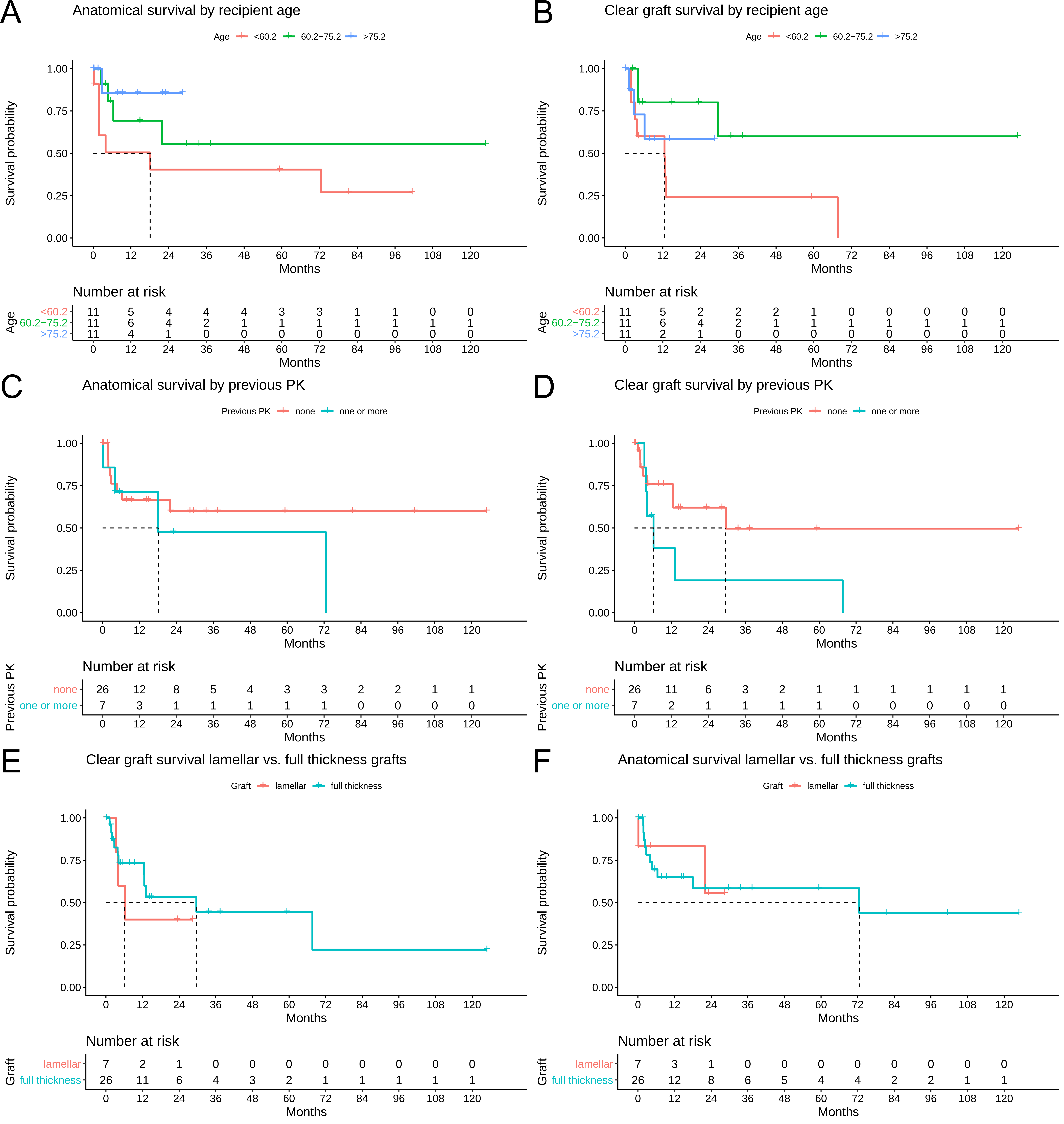
A
Anatomical survival by recipient age
Age <60.2 60.2−75.2 >75.2
Survival probability
1.00 0.75 0.50 0.25 0.00
0 12 24 36 48 60 72 84 96 108 120
Months
Number at risk
Age
<60.2 11 5 4 4 4 3 3 1 1 0 0
60.2−75.2 11 6 4 2 1 1 1 1 1 1 1
>75.2 11 4 1 0 0 0 0 0 0 0 0
0 12 24 36 48 60 72 84 96 108 120
Months
B
Clear graft survival by recipient age
Age <60.2 60.2−75.2 >75.2
Survival probability
1.00 0.75 0.50 0.25 0.00
0 12 24 36 48 60 72 84 96 108 120
Months
Number at risk
Age
<60.2 11 5 2 2 2 1 0 0 0 0 0
60.2−75.2 11 6 4 2 1 1 1 1 1 1 1
>75.2 11 2 1 0 0 0 0 0 0 0 0
0 12 24 36 48 60 72 84 96 108 120
Months
C
Anatomical survival by previous PK
Previous PK none one or more
Survival probability
1.00 0.75 0.50 0.25 0.00
0 12 24 36 48 60 72 84 96 108 120
Months
Number at risk
Previous PK
none 26 12 8 5 4 3 3 2 2 1 1
one or more 7 3 1 1 1 1 1 0 0 0 0
0 12 24 36 48 60 72 84 96 108 120
Months
D
Clear graft survival by previous PK
Previous PK none one or more
Survival probability
1.00 0.75 0.50 0.25 0.00
0 12 24 36 48 60 72 84 96 108 120
Months
Number at risk
Previous PK
none 26 11 6 3 2 1 1 1 1 1 1
one or more 7 2 1 1 1 1 0 0 0 0 0
0 12 24 36 48 60 72 84 96 108 120
Months
E
Clear graft survival lamellar vs. full thickness grafts
Graft lamellar full thickness
Survival probability
1.00 0.75 0.50 0.25 0.00
0 12 24 36 48 60 72 84 96 108 120
Months
Number at risk
Graft
lamellar 7 2 1 0 0 0 0 0 0 0 0
full thickness 26 11 6 4 3 2 1 1 1 1 1
0 12 24 36 48 60 72 84 96 108 120
Months
F
Anatomical survival lamellar vs. full thickness grafts
Graft lamellar full thickness
Survival probability
1.00 0.75 0.50 0.25 0.00
0 12 24 36 48 60 72 84 96 108 120
Months
Number at risk
Graft
lamellar 7 3 1 0 0 0 0 0 0 0 0
full thickness 26 12 8 6 5 4 4 2 2 1 1
0 12 24 36 48 60 72 84 96 108 120
Months
