# Supplementary figures and images for: Indications, techniques, and graft survival of mini and corneo-scleral tectonic keratoplasties: A retrospective single-center case series
Source: PLoS One. 2023 Aug 4;18(8):e0289601. doi: 10.1371/journal.pone.0289601 (PMC10403125; doi:10.1371/journal.pone.0289601)

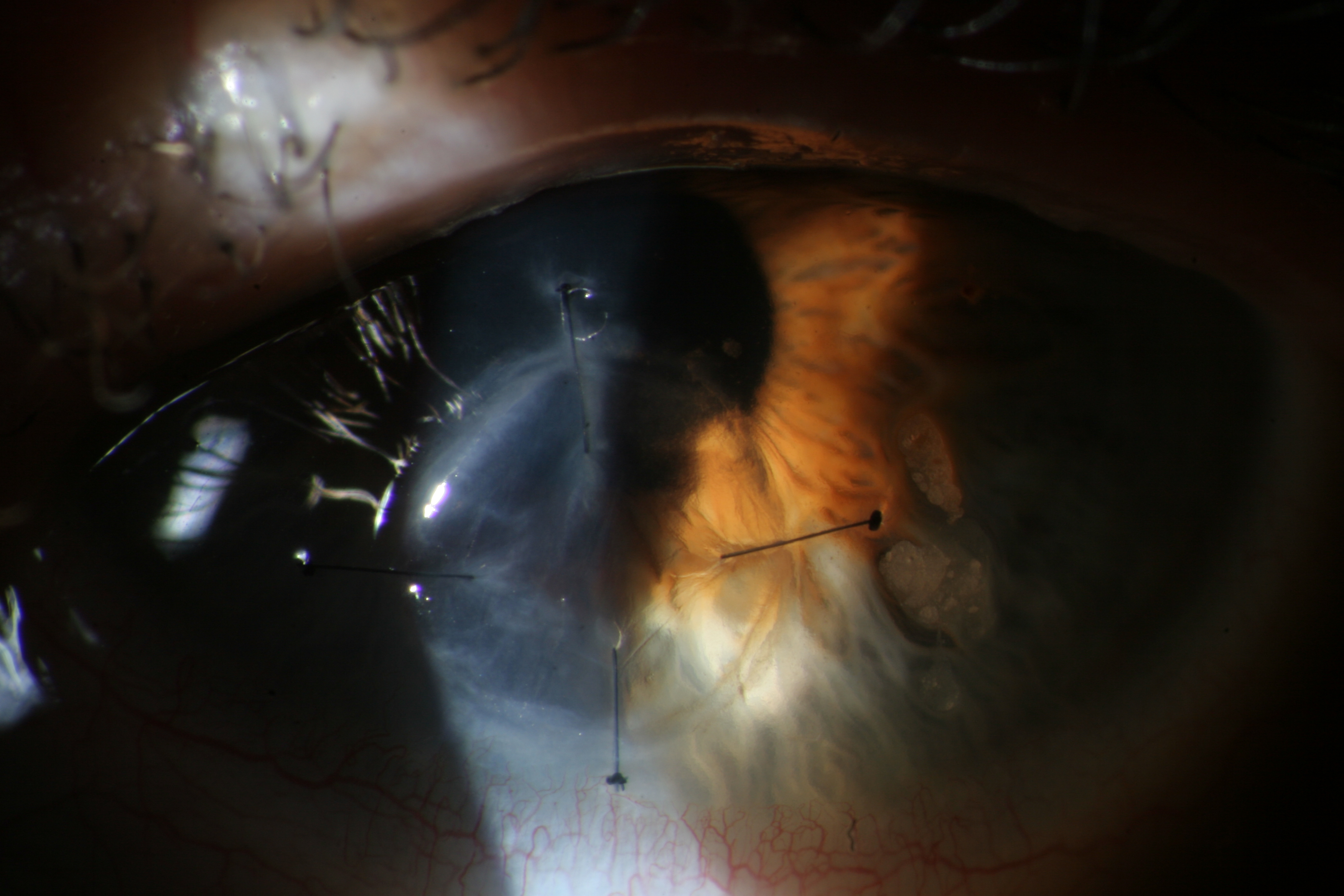

Supplement: S2 Fig — (JPG) [file pone.0289601.s002.JPG]

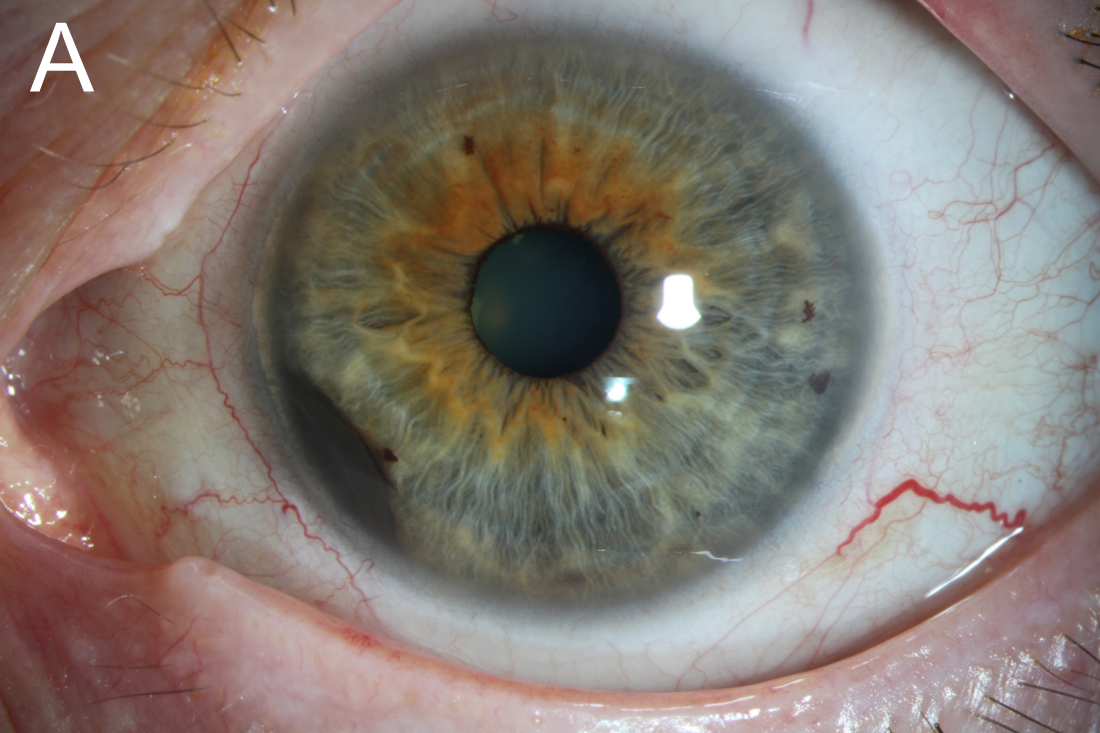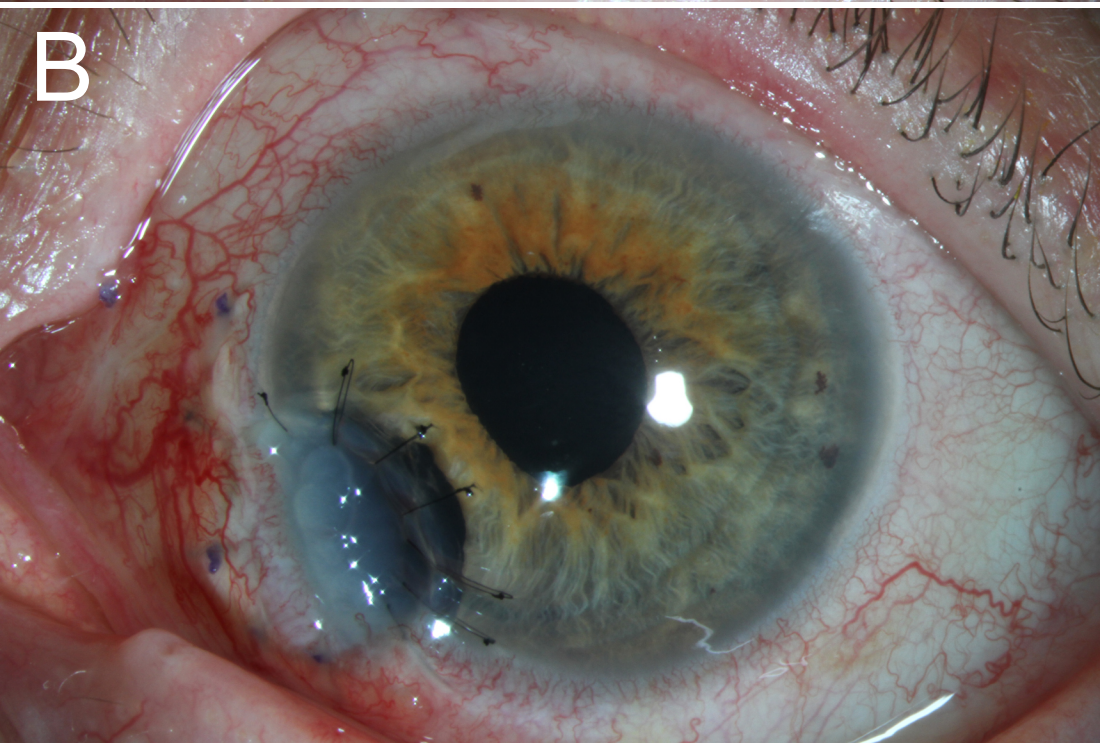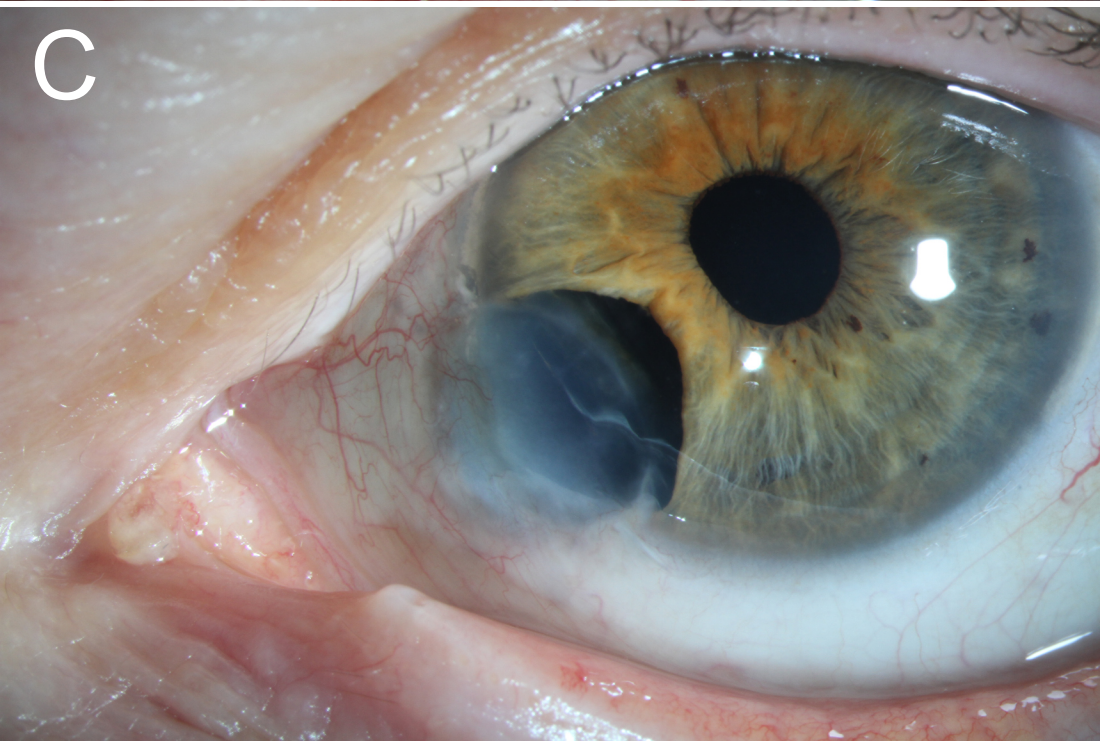

Supplement: S3 Fig — Patient ID 28 A: Patient with ciliary-body/iris-melanoma one month and 22 days pre-surgery, B: 27 days post-surgery, C: Three years and four months post-surgery. (PDF) [file pone.0289601.s003.pdf]

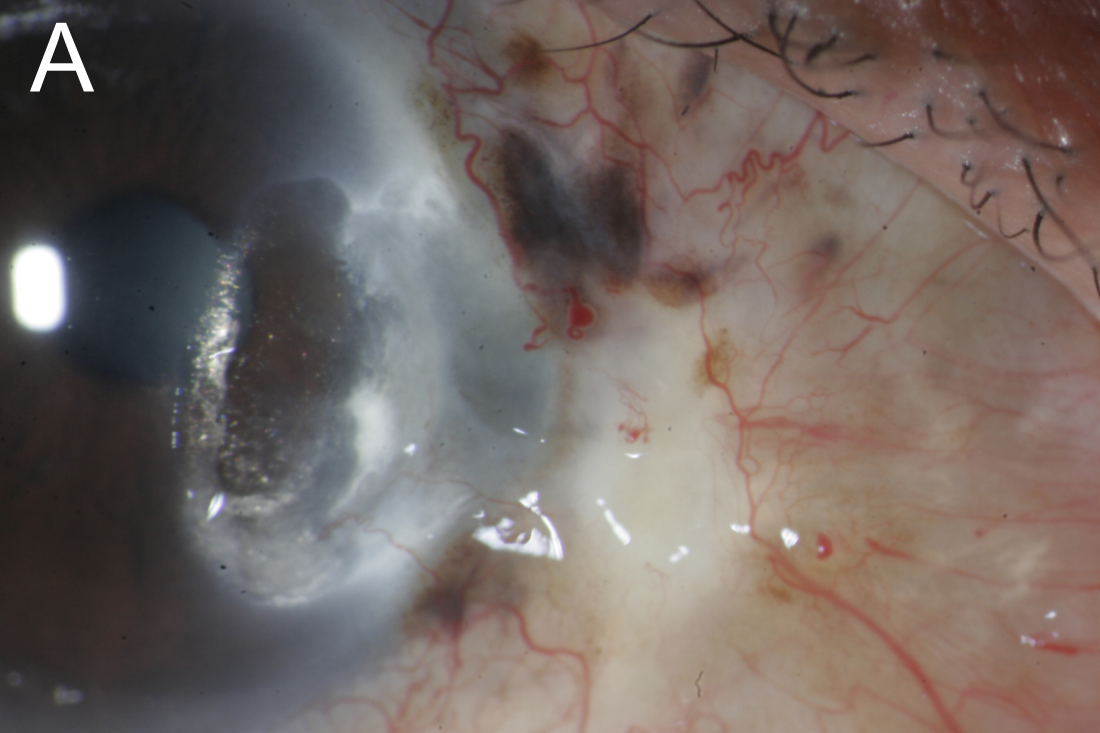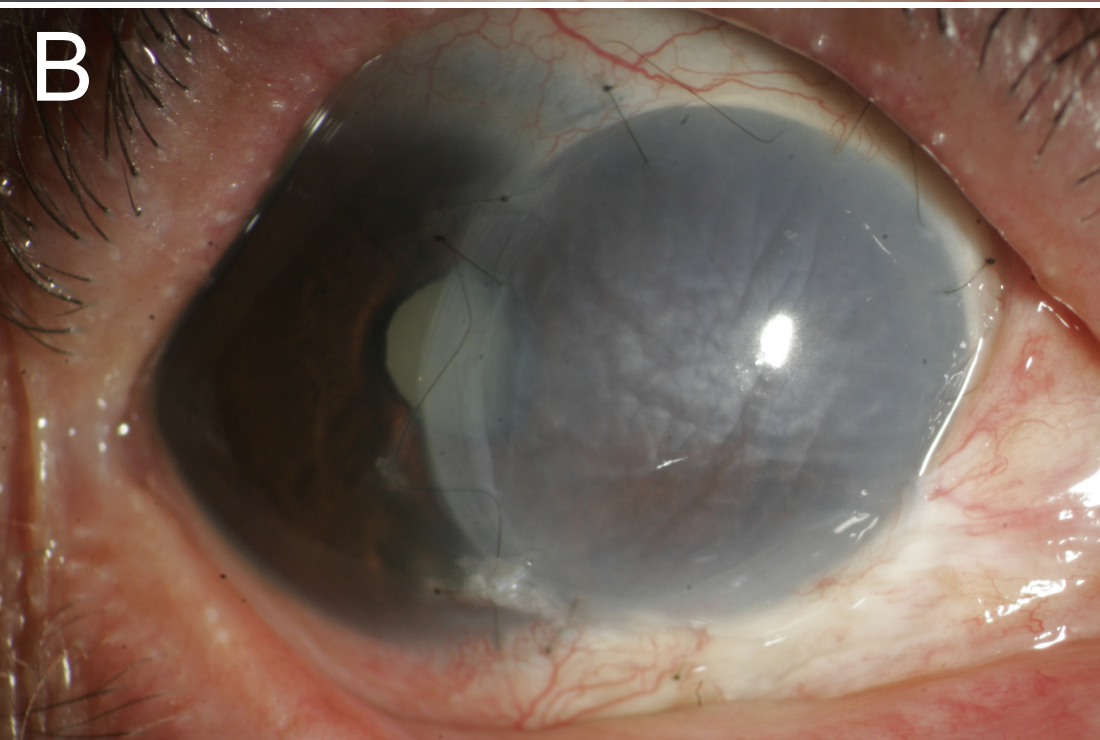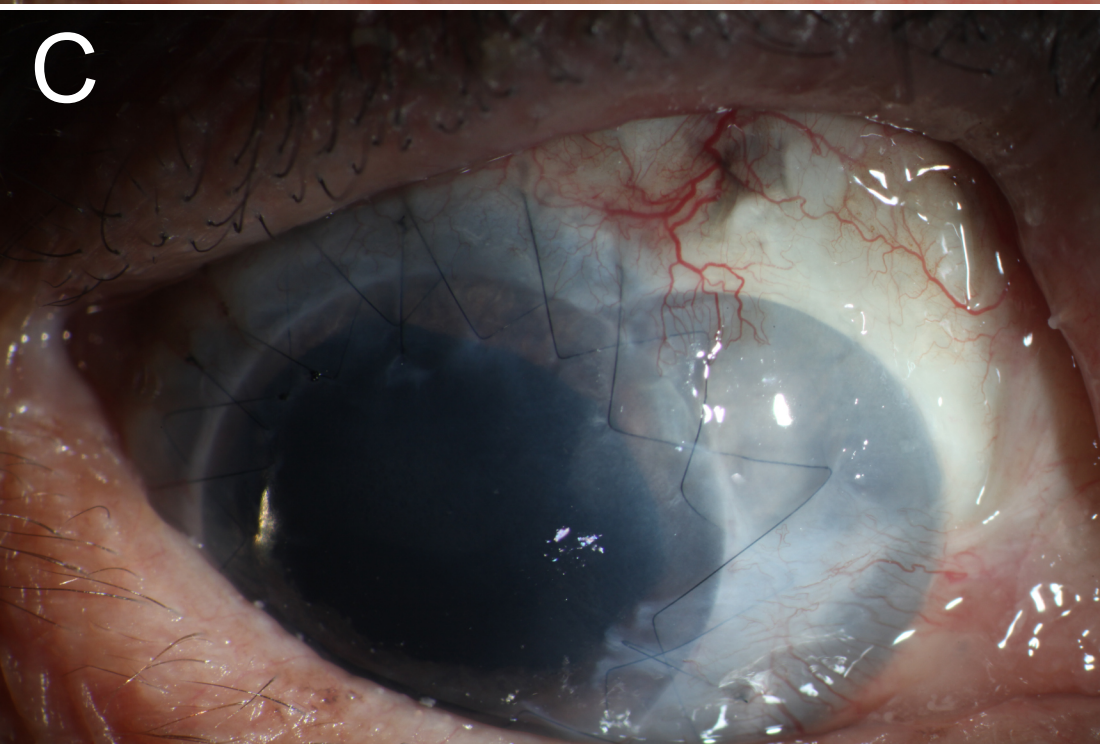

Supplement: S4 Fig — Patient ID 9 A: 20 days pre-surgery, presenting conjunctival melanosis, corneal thinning and deep corneal vascularization after radiation of a pigmented tumor in this area 40 years before at a different hospital. B: 4 months post-surgery, C: Three years and five months after initial tectonic keratoplasty and after repeated central keratoplasty because of recurrent corneal ulcer. (PDF) [file pone.0289601.s004.pdf]

A

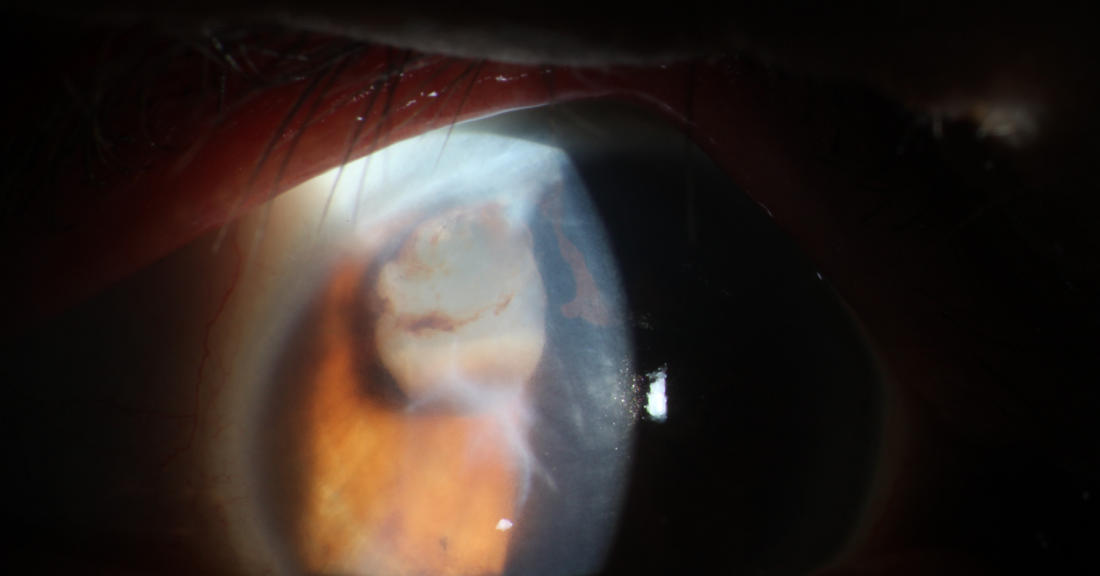

B

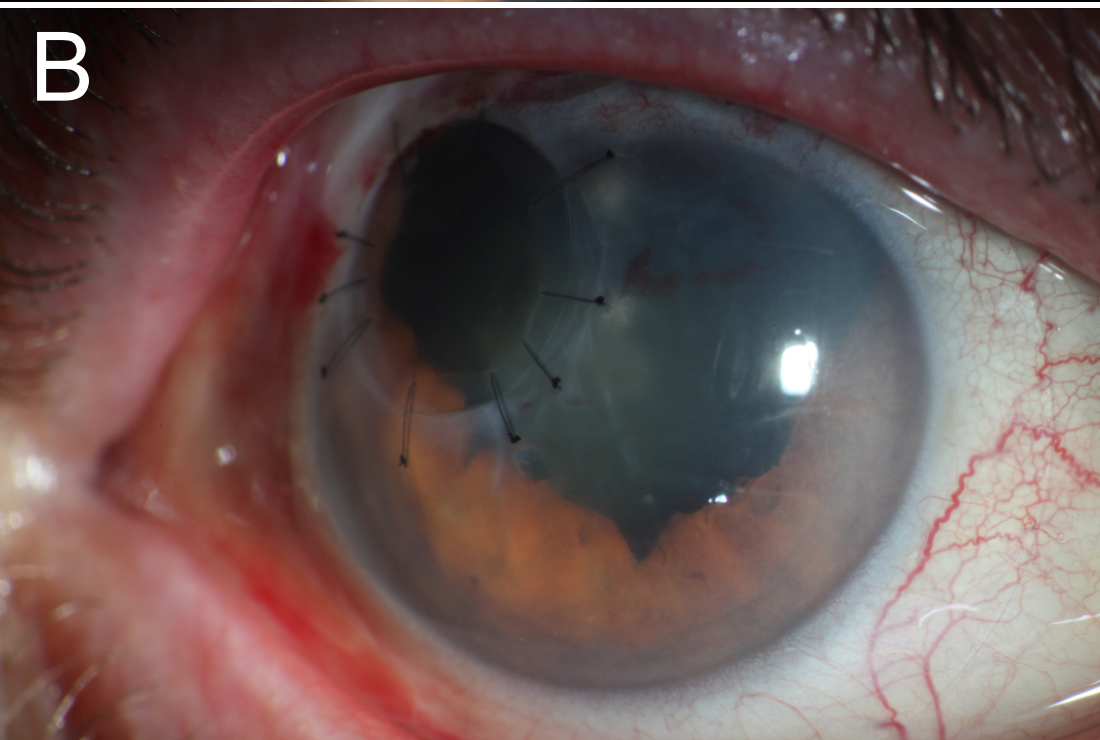

C

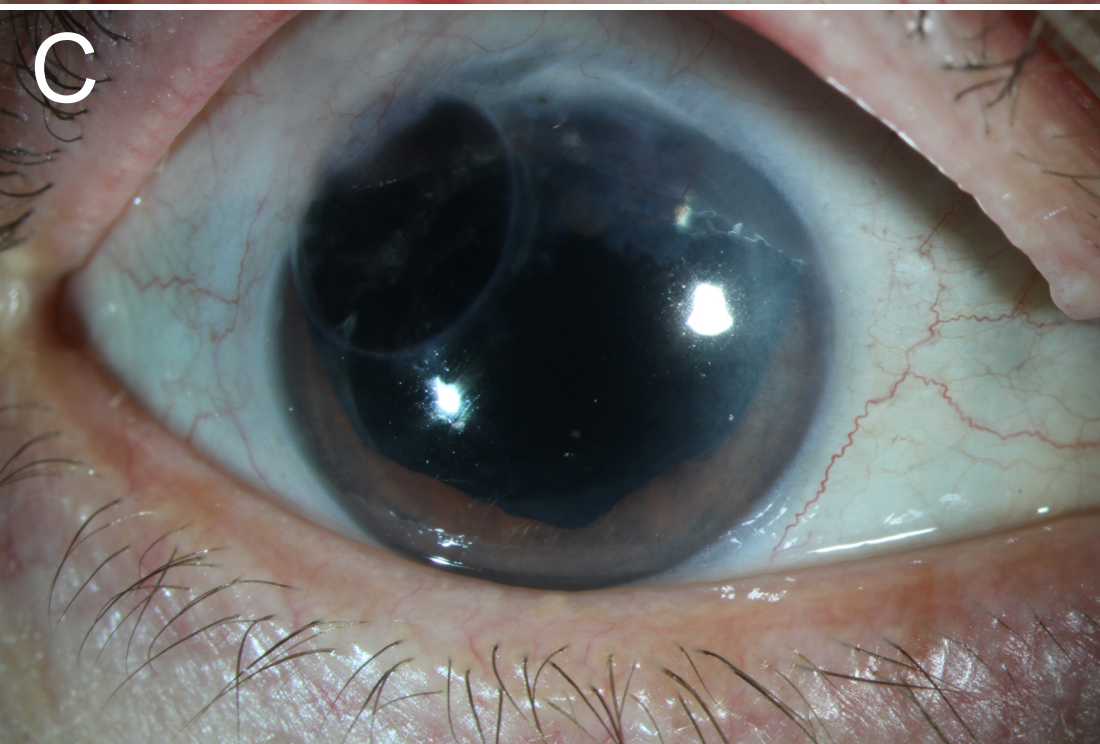

Supplement: S5 Fig — Patient ID 15 A: Pre-surgery: Epithelial downgrowth with cystic formation after explosive trauma as a child, B: One day post-surgery, C: Six years post-surgery. (PDF) [file pone.0289601.s005.pdf]
