# Supplementary material for: Indications, techniques, and graft survival of mini and corneo-scleral tectonic keratoplasties: A retrospective single-center case series
Source: PLoS One. 2023 Aug 4;18(8):e0289601. doi: 10.1371/journal.pone.0289601 (PMC10403125; doi:10.1371/journal.pone.0289601)
Supplement: S1 Table — ID 19 and 24 correspond to the right and left eye from the same patient. The width of the blue bar at the columns age at surgery, diameter and follow up (FU) are proportional to the numbers displayed. In the endothelial cell density (ECD) column, yellow represents low and blue high ECD. (DOCX) [file pone.0289601.s007.docx]

| S1 Table: Individual patient and clinical characteristics of all included patients at first TK at our eye center. ID 19 and 24 correspond to the right and left eye from the same patient. The width of the blue bar at the columns age at surgery, diameter and follow up (FU) are proportional to the numbers displayed. In the endothelial cell density (ECD) column, yellow represents low and blue high ECD. |
| --- |
